# Women's Attitudes and Health Beliefs toward Osteoporosis Screening in a Community Pharmacy

**DOI:** 10.1155/2013/650136

**Published:** 2013-05-28

**Authors:** Priyanka Deo, Rajesh Nayak, Jigar Rajpura

**Affiliations:** ^1^Department of Pharmacy Administration, College of Pharmacy and Health Sciences, St. John's University, 8000 Utopia Parkway, Jamaica, NY 11439, USA; ^2^Department of Pharmacy Practice, College of Pharmacy, Purdue University, 575 Stadium Mall Drive, West Lafayette, IN 47907, USA

## Abstract

The aim of this study was to examine women's attitudes and health beliefs towards osteoporosis screening in a community pharmacy setting, utilizing the theoretical framework of Health Belief Model. A nonexperimental, cross-sectional research design, examining a convenience sample of women aged 18 and over, from several New York City senior care centers, a church, and a university campus in New York, was employed to assess the study objectives. Osteoporosis Health Belief Scale questionnaire was used to study the attitudes and health beliefs of participants towards bone mineral density screening in community pharmacy. From the study, it was observed that perceptions of severity and susceptibility towards osteoporosis and subjects' demographic characteristics did not seem to significantly influence the decision to screen in a community pharmacy setting. The perceptions of benefits of community pharmacy-based osteoporosis screening and the perceived barriers were found to be of greater importance in women's decisions to engage in osteoporosis-specific preventive behavior.

## 1. Introduction

Osteoporosis is an asymptomatic skeletal disorder, referred to as the “silent disease.” As a result, people fail to realize having osteoporosis, until a sudden fall or strain causing the bones to fracture or vertebral structure to collapse. According to the National Osteoporosis Foundation (NOF), osteoporosis poses a major public threat to an estimated 44 million Americans or 55% of the people 50 years of age and older. Statistics reveal that 80% of the individuals suffering from osteoporosis are women. In American females, the incidence of a fracture is estimated to be 5.5 times greater than that of breast cancer, 4.4 times more prevalent than stroke, and twice the yearly incidence of heart attacks [1]. The direct care expenditures for osteoporotic fractures alone range from $12.2 to 17.9 billion each year, measured in 2002 US dollars [2]. The negative outcomes of the disease can be fractures, crippling, and even death. Furthermore, osteoporosis results in reduced quality of life, avoidance of social interaction due to low self-esteem, physical pain in activities of daily living, emotional suffering, anxiety regarding the fear of fracture, and depression about being dependent on others (Bone Health and Osteoporosis: A Surgeon General's Report [3]). Awareness regarding osteoporosis is increasing among women, owing to the increased media exposure and educational campaigns by the public and private healthcare organizations. Evidence, however, shows that increased awareness does not necessarily translate to appropriate preventive behaviors [4, 5]. Lack of concern and knowledge can be attributed to lower perceptions of susceptibility [5, 6] and severity of the disease [7]. The rationale for the use of a BMD testing lies in the assumption that the lower the bone density the greater the risk of osteoporosis-related fracture. Since osteoporosis is asymptomatic, early detection of the risk level for a person can help monitor wear and tear of bones and be instrumental in lowering the incidence of the disease through appropriate lifestyle modifications (diet and exercise) and medication use. BMD testing coupled with physical assessment and identification of risk factors is the only way of identifying individuals at risk for osteoporosis. NOF, US Preventive Services Task Force (USPSTF), and the American College of Obstetricians and gynecologists Committee on Gynecologic Practice (ACOG) recommend BMD testing for all women aged 65 and older regardless of risk factors and younger women with certain characteristic risk factors. The American Association of Clinical Endocrinologists (AACE) also recommends testing for premenopausal women with risk factors for fractures. The screening can be used to assess fracture risk and to establish the diagnosis and severity of osteoporosis (National Osteoporosis Foundation: About Osteoporosis, 2005). In addition, it can also used to assess changes over time in those individuals on medication therapy compared to those who are not. It might be also be instrumental in influencing an individual's treatment decision and can lead to behavioral changes. However, according to the USPSTF, there is insufficient evidence to recommend for or against any routine osteoporosis and screening in postmenopausal women who are younger than 60 or in women aged 60–64 who are not at an increased risk for osteoporotic fractures. This raises the question whether women between the ages of 18 and 35 should consider screening for osteoporosis when still building their bone mass because an osteopenic female over 18 years of age will become osteoporotic by the time she turns 50 or 60 years of age. Furthermore, premenopausal osteoporosis can also occur because of chronic glucocorticoid therapy, prolonged amenorrhea, anorexia nervosa, rheumatoid arthritis, and diseases that affect calcium and vitamin D metabolism, thus underscoring the importance of prompt screening in such cases. It is also advised that females over the age of 30 should get a good baseline measurement of bone density before they enter menopause, since women can lose bone very rapidly during and after menopause. This is substantiated by the results from a study [1] wherein several 30-year-old women were found to be osteopenic when screened for osteoporosis, proving that there is an increased need for continuous assessment of bone density and preventive measures. Furthermore, an analysis of peripheral BMD and fractures in over 200,000 women suggests that half of all fragility fractures occur in females with low bone mass [8]. 

The preceding review of the literature suggested that women who have osteoporosis are generally unaware of their skeletal status [9, 10]. This lack of recognition can be attributed to the high cost of screening and limited access to bone density measuring devices. Moreover, screening tests are only valuable if their results influence decisions about treatment. Some studies have also shown that bone mineral density measurement leads to behavioral changes in women. One study reported that women with knowledge of their own low bone density levels were more likely to change their health behavior to prevent fractures than women with normal bone density levels [11, 12]. Another study reported that receiving a bone density screening increased the perceived susceptibility of the participants who had a low BMD [7]. According to Cook et al. (1991), a significant increase in the knowledge levels and understanding of osteoporosis among women led to behavioral changes in participants who had undergone screening after one year [13]. It was also found that a high proportion of well-insured women with higher education and with below-normal BMD were mostly the ones to have made lifestyle changes [13]. Results from other studies also show that receiving a BMD test influenced preventive behavior among participants who received the screening [14, 15]. One study reported that screening for osteoporosis was associated with statistically significant lower hazard of hip fractures [16]. In sum, all the aforementioned studies substantiate the fact that BMD screening is an effective and essential tool in diagnosing osteoporosis and might be instrumental in influencing an individual's treatment decision and leads to behavioral changes. 

Community pharmacies are in an excellent position to help meet the need for broader testing of the population by providing the measurement of peripheral bone density with inexpensive portable units. Pharmacies offering bone density screening services generally utilize peripheral SXA, DXA, and QUS methods to measure BMD. These devices measure BMD at sites such as the hand, wrist, and heel. Osteoporosis screening in community pharmacies is a unique way of shifting the pharmacist's focus from dispensing to patient-centered care. Evidence shows that pharmacists are in an excellent position to help patients gain compliance with and manage their medication therapy related to osteoporosis care [1, 10, 17]. Benefits of BMD screening in a pharmacy include convenience to the patient with regard to store location, store hours, and easy accessibility to the pharmacist and opportunities for improving patient-physician relationship. The cost of screening is not a major factor in decisions to obtain screening in the community pharmacies, as previous studies have shown that pharmacies charge only about $25–$50 for providing osteoporosis-related screening services [1, 10, 18] and that participants are often willing to pay $15–$66 for such services [1, 17, 19]. Most of these studies examining the effectiveness of pharmacist-provided osteoporosis screening services have looked at the impact of BMD screening on patient outcomes, specifically their decisions related to healthcare, lifestyle modifications, use of over-the-counter and prescription medications, and their communication with the physician [9, 10, 17]. The feasibility (economic and other) of providing such service, [9, 17] and physician acceptance of these programs [9] have also been reviewed in the literature. However, the decision to receive peripheral bone density testing can be beneficial only if it leads to positive behavioral changes in the patient with regard to the self-management of osteoporosis. A review of the literature shows that knowledge by itself is not a strong enough predictor of intention to take a preventive action [4, 6] and sociodemographic factors are a poor predictor of intention as well [20]. Therefore, it is important to consider, in addition to the sociodemographic factors, the influence of other socio-psychological variables. The Health Belief Model (HBM), that was developed in the 1950s, consists of several constructs that explain the theoretical links between disease perceptions such as perceived susceptibility to the disease, perceived severity of the disease, health beliefs such as perceived benefits of taking an action as compared to the perceived barriers of engaging in an action, and the likelihood of engaging in a preventive behavior. That is, according to the postulates of the well-known HBM [21, 22], the individual should also believe that the disease is serious enough, such that he/she is susceptible to it and that the benefits of taking a particular action exceed the barriers. However, research published to date has focused little attention on the patient preferences for participating in an osteoporosis-screening program and beliefs related to their health condition. For example, there is lack of information about how frequently women screen for osteoporosis and whether demographics such as age, education, ethnicity, and preferences for screening play a prominent role in women's decision regarding screening for the disease. Furthermore, it is not known whether beliefs regarding the disease itself and attitudes toward screening would have any influence on the patient's decision to participate in a screening program. Therefore, it was the objective of this study to make an assessment of women's health beliefs about and attitudes toward BMD screening, particularly screening programs available in a pharmacy and their intention to obtain such screening by utilizing the conceptual framework of the HBM. 

## 2. Objectives 

The goal of this study was to examine women's health beliefs regarding osteoporosis and their perceptions related to screening for the disease, in a community pharmacy setting, by utilizing the theoretical framework of HBM. The study also evaluated the role of demographic factors in shaping women's beliefs about osteoporosis and assessed possible associations between demographic characteristics with the preventive behavior of interest (i.e., screening for osteoporosis in a community pharmacy), based on the conceptualization of the HBM. 

## 3. Methods 

### 3.1. Survey Description

The study employed a nonexperimental research design utilizing convenience-sampling strategies for recruiting the participants. According to NOF, women form the majority (about 80%) of the population suffering from osteoporosis (National Osteoporosis Foundation: About Osteoporosis, 2005); thus, women aged 18 and above, without any prior diagnosis of osteoporosis or on a medication therapy for osteoporosis, were considered eligible to participate in the study. The study participants were required to fill out a self-administered questionnaire, which had been appropriately pretested and approved by the Institutional Review Board (IRB) at St. John's University, Jamaica, NY, USA. The survey was conducted at three senior care centers, a church located in Queens County, New York, and the St. John's University campus in June 2005. 

### 3.2. Survey Instrument

Self-administered questionnaire based on the postulates of the HBM was used for the study. Most of the study's objectives were addressed by utilizing a previously developed OHBS, which represented various components of the HBM. The 42-item OHBS developed by [23] is based on the theoretical framework of HBM, which assesses the health beliefs of people regarding osteoporosis. The OHBS consists of seven subscales: severity, susceptibility, health motivation, calcium benefits, calcium barriers, exercise barriers, and benefits. The subscales measuring the concepts of barriers and benefits are specific to calcium intake and exercise behavior only and are different for the two scales addressing calcium intake and exercise level. Items 1–6 in the questionnaire address susceptibility; items 7–12, the severity; items 13–18, the benefits of exercise; items 19–24, the benefits of calcium intake; items 25–30, the barriers to exercise; items 31–36, the barriers to calcium intake; and items 37–42, the health motivation. Response for each item in seven subscales ranges from “strongly disagree” (1 point) to “strongly agree” (5 points). Since there are six items in each subscale, the potential score range for each subscale is 6 to 30, for a total possible score of 42 to 210 for the entire scale. Reference [23] reported a reliability of 0.74 for the entire scale at pretest and 0.84 at posttest in a study sample of 150 elderly subjects, a majority of whom were women in the age range of 60–93 years. 

In order to address questions relevant to the study at hand, and to incorporate beliefs about benefits, and understand the barriers of pharmacy-based testing, the instrument was slightly modified to measure women's perceptions of BMD screening in a community pharmacy. Scale modification included deletion of the subscales of benefits and barriers related to calcium (12 items) and exercise (12 items). These were replaced instead by statements evaluating benefits and barriers (8 items each) specific to osteoporosis screening in a community pharmacy. The items related to susceptibility (6 items), severity (6 items), and health motivation (six items) were retained with no further modification. A brief description explaining BMD screening in a pharmacy was also incorporated in the questionnaire for the benefit of women unfamiliar with the procedures of pharmacy-based screening. A screening question was used to determine the subjects' inclusion in the study. For this purpose, the questionnaire started with a short statement asking only those female respondents, who were 18 years of age and over, to fill out the survey, thus ensuring that only the appropriate study sample was selected. A preliminary question asking the respondents whether they were previously diagnosed with osteoporosis or were currently on a medication therapy for treating osteoporosis was also added to the survey. This question ensured that women who have received osteoporosis screening in the past were screened out. 

### 3.3. Pretesting of the Questionnaire

The readability of the questionnaire was initially established by a panel of expert judges consisting of faculty at the Department of Pharmacy and Administrative Sciences at St. John's University. Three graduate students at the department were also asked to review the instrument for errors, poorly worded, and misleading and/or confusing items. In addition, the questionnaire was pretested on two elderly women: one undergraduate student and two middle-aged women on St. John's University Campus to determine any ambiguity in the understanding of questions. Content validity for the modified questionnaire was established by the faculty at the College of Pharmacy, St. John's University, three graduate students, and five women belonging to different age groups. The questionnaire was also tested for face validity to see whether each of the eight newly added items appeared to measure what represented the benefits of and barriers to pharmacy-based testing and to check their appropriateness for inclusion in the survey. 

### 3.4. Data Collection Procedure

At the senior centers and the church, flyers announcing the administration of the surveys on specific days were put up, a week prior to the survey at the survey sites. Because all the senior centers were similar in their organization, an identical approach was adopted for the survey administration at these sites, with the exception of the university site where a relatively different procedure was employed for subject recruitment. At the university site, surveys were distributed to the staff of the College of Pharmacy, the university library, the registrar's office, and the Athletics Department. These were later collected after a period of 7 days. At the senior care centers, efforts were made to ensure uniformity in the survey administration by distributing the surveys to an assembled group of elderly females, following the IRB guidelines with respect to participant confidentiality. Participants were assured of total confidentiality, and recruitment process was entirely based on voluntary participation. Subjects were required to sign the consent form in order to participate in the study. The surveys were coded numerically for data entry purposes, and a note was made of the number of surveys distributed at each site.

## 4. Results 

Of 172 questionnaires that were distributed at five different study sites, 117 were collected (Table 1). Of these, 109 usable questionnaires were retained after eliminating eight questionnaires (three questionnaires were found to be incomplete and five participant questionnaires were found to have had a prior diagnosis for osteoporosis). Of the 109 questionnaires selected, a variable specific computed, series mean, was used to replace missing values. None of the 109 usable questionnaires had more than 15% of the total items with missing values. An overall response rate of 68.02% was achieved using the convenience sampling strategies outlined. Following data collection, responses from the 109 questionnaires were coded and scored, and the data was entered into the database for further analyses. An exact 95% CI was calculated when appropriate. All statistical analyses were performed using SPSS version 12.0 (SPSS Inc., Chicago, IL, USA). A *P*-value < 0.05 was regarded as statistically significant. Posttest reliability analysis for the individual OHBS subscales revealed Cronbach's alpha reliability coefficients to range from 0.799 (perceived severity) to 0.910 (perceived susceptibility) for a sample of 109 females, 18 years of age and over. The study sample included 109 females 18 years of age and over, recruited from various study sites across the metropolitan New York, USA. A bulk of the sample comprised of Caucasians (56.9%) and about 20% represented Asians (Table 2). A majority of the respondents (*n* = 41) were in the age group of 18–30 years and accounted for 37.6% of the sample, while females over 60 years of age accounted for nearly a quarter of the sample (24.8%). Of the 109 respondents, the majority (33%) had a graduate degree, 28.4% had done some college work, and 24.8% had an undergraduate degree. About 46% of the females were married while 39.4% were single. 67% of the respondents had private insurance, and the rest either had Medicare (*n* = 14), Medicaid (*n* = 6), or Medicare plus coverage (*n* = 6). About 71% had heard about bone mineral density testing prior to this survey. Only about 9% had received a BMD test in a pharmacy before (with no prior diagnosis of osteoporosis). 

### 4.1. Data Analysis

 The study examined the hypothesis that a woman's intention to screen in a pharmacy would depend on her attitudes toward pharmacy-based screening for osteoporosis. Pearson's correlation coefficient was used to test the association between intention to screen and attitudes toward screening in a pharmacy. The results (Table 3) from the analysis showed that a strong positive correlation (*r* = 0.553, *P* = 0.000) existed between a woman's intention to screen for osteoporosis in pharmacy and her attitudes toward screening in a community pharmacy, thus confirming the first research hypothesis. 

 Further, a hypothesis stating that the more severe the perception of severity of and susceptibility to osteoporosis the greater the likelihood of screening in a pharmacy, exhibited a weak positive correlation between perceptions of severity of and susceptibility to osteoporosis and the intention to screen in a pharmacy (*r* = 0.205, *P* = 0.033 and *r* = 0.157, *P* = 0.102, resp.) (Table 3). However, association between the perceptions of susceptibility and screening intentions were not statistically significant (Table 3). 

 Following the testing of the research hypotheses, further analyses revealed a positive and significant correlation (*r* = 0.311, *P* = 0.001) between the perceived benefits of pharmacy-based osteoporosis screening and intention to screen for osteoporosis (Table 3), while the perceived barriers to osteoporosis screening in a pharmacy and the likelihood of screening were negatively correlated (*r* = −0.294, *P* = 0.002) with each other as expected (Table 3). In addition, the health motivation scores for each respondent were found to correlate weakly with the intention to screen for the sample surveyed (*r* = 0.033, *P* = 0.734) (Table 3). 

 Second objective of the study investigated whether sociodemographic factors of the study participants were associated with the intention to screen in a pharmacy. For this purpose, women who responded to the 5-point intention scale positively (i.e., “very likely” and “likely”) and negatively (“very unlikely” and “unlikely”) were separated into two groups and women responding with neutral (do not know) were excluded from the analysis. Thus, there were 44 (40%) women who thought they were likely to some degree to visit a pharmacy for screening in the future and 36 (33%) women who did not intend to screen in a pharmacy. Chi square association statistic was computed for the two groups with respect to age, ethnicity, education, marital status, and insurance coverage (Table 4). No significant association was found between the respondent's ethnicity, education, marital status, insurance coverage, and the likelihood of screening for osteoporosis in a community pharmacy (Table 4). However, the study found that respondent's age was significantly related to the likelihood of screening. 

 Analysis of variance (ANOVA) (Table 5) showed no significant overall group differences on the perceptions of susceptibility, seriousness, benefits, barriers, and health motivation towards pharmacy-based testing for the three age groups. However, attitudes toward pharmacy-based testing differed significantly for women belonging to the age group of 31–60 years and those over 60 years of age (*P* = 0.000). 

## 5. Discussion 

The study findings revealed that about 71% of the sample had heard of BMD testing prior to the survey, but only a few had awareness regarding pharmacy-based testing for osteoporosis. This finding is similar to the previous findings [20], which reported a substantial awareness among women about osteoporosis in general. This awareness may probably be attributed to increased exposure to media disseminating information about this medical condition and informational campaigns about osteoporosis conducted by the public and private healthcare organizations across the country. Despite the substantial awareness regarding BMD testing in general, the awareness regarding pharmacy-based testing in particular seems to be limited among the population surveyed. The reasons for this finding are currently unknown. Pharmacy-based osteoporosis screening is relatively a more recent development in pharmacy practice, and the public knowledge of this new service is limited to only regions where pharmacists are known to provide these services. In New York, it can be argued that the profession has yet to make strides in the creation of awareness of this new service as this service is not widely offered by New York pharmacies.

 Study findings provide further insights into the beliefs and attitudes that might be crucial for shaping women's health behavior. Upon examining the relationship between women's attitudes toward pharmacy-based osteoporosis screening and the intention to screen in a pharmacy, results indicated a significant positive association (*r* = 0.553, *P* = 0.000). An implication of this finding may be that attitudes toward a disease may play a crucial role in the prediction of an individual's health behavior. A research question developed to examine whether the perceived benefits and barriers of pharmacy-based screening influenced women's screening decisions exhibited a significant positive association between the perceived benefits of screening in a pharmacy and the likelihood of screening in the future. The results also indicated that the majority of the surveyed women perceived effective management of osteoporosis-related medication therapy, improvement in exercise levels, and overall prevention of osteoporosis as the major benefits of pharmacy-based testing which may play a role in their decision to screen. Furthermore, perceptions of benefits were also found to positively and significantly correlate with the attitudes toward pharmacy-based screening. Factors like concern about physician approval of the pharmacy testing, cost of screening, anxiety about pharmacy-based testing, and so forth, were found to have a negative association with screening decisions, but the magnitude of the correlation was statistically insignificant. The study also found negative associations between the decision to screen and the beliefs that a pharmacy was not an appropriate setting for BMD screening, that a pharmacist was not the right person to perform screening, and that a pharmacy offered little privacy. This negative association points to a probable reason for the previously documented evidence of lower screening rates for bone density in community pharmacies [9]. Furthermore, it points to a common misperception of the pharmacy profession by most people. As documented by [24], individuals generally consider doctors to be a more appropriate source of care giving than pharmacists. Besides, pharmacists are most often thought of as only dispensers of medications. Therefore, it can be argued that women's perceptions about the barriers to pharmacy-based testing perhaps have a stronger influence on their screening decisions. It is evident from the results of the analysis that changing perceptions related to privacy and the impression of pharmacists as just pill dispensers could help in promoting BMD screenings in pharmacy. 

It was observed from the data that the more severe the perceptions of severity of and susceptibility to osteoporosis the greater the intention to screen for osteoporosis in a pharmacy. Based on the precepts of the HBM, postulating that perceptions of susceptibility and severity are helpful in explaining an individual's health behavior stands true. The results from the data analysis showed that there was no significant association between the aforementioned perceptions of susceptibility and the intention to screen for the disease in a pharmacy. Furthermore, the magnitude of positive correlation that was found between the perceptions of severity and the likelihood of screening though statistically significant was very small. The perceptions of severity and susceptibility to osteoporosis did not influence women to screen for osteoporosis in a pharmacy. As in the previous studies on preventive health behavior, perceived severity was unrelated to intention. It was not surprising that perceived susceptibility, which has been documented in a lot of studies as an important HBM dimension [25], was found to have no correlation to intention, since previous studies on osteoporosis specifically utilizing the HBM have reported a very low perception of susceptibility to the disease among most women [5–7, 20, 26]. The reasons for this low perception of susceptibility and severity to osteoporosis among the general population are currently unknown. A careful examination of participant responses to individual questionnaire items further revealed that many women responded with “neutral” to the questions evaluating their perceptions of susceptibility to osteoporosis based on certain risk factors such as their body build and family history in particular and their chances of getting osteoporosis in general. For example, analysis of the comments made by the study participants, such as “My grandmother has osteoporosis, but I do not know if that makes me susceptible to osteoporosis” or “I wouldn't know what risk factors would cause osteoporosis” indicate that women lack knowledge about the disease. It is possible that knowledge about osteoporosis could increase the perceived susceptibility and severity to osteoporosis, and this relationship needs to be explored further. The implication that follows from the foregoing discussion is that women in general display more awareness about the disease, but also appear to lack knowledge about the disease, despite widespread exposure to information about this medical condition. 

The association of sociodemographic factors and the intention to screen for osteoporosis in a pharmacy showed no significance between ethnicity, education, marital status, insurance coverage, and the intention to screen. However, age was found to be significantly related to women's screening intentions. These findings are partly consistent with the findings of [26], which reported that sociodemographics such as age and education were associated with the intention to prevent osteoporosis in women. However, the results differed from those of [20], which reported that demographic factors such as age, race, or education did not influence osteoporosis preventive behavior in women. Therefore, given the conflicting evidence from the past research, no conclusive remarks may be drawn now based on these findings alone. More research is needed in this area, especially since age and ethnicity are shown to be two major risk factors of osteoporosis. 

## 6. Limitations 

The generalizability of the study sample may be limited as it consisted of individuals recruited that represented a small geographic region within New York City. The generalizability of the research findings to a larger New York population may also be limited because of a possible underrepresentation of ethnic minorities in our sample. Further, a convenience sampling methodology was used to collect data. As a result, there may have been some volunteer bias in the results obtained. Because the respondents were not randomly sampled or selected, statistical results have to be viewed with caution, as representative normal distributions were not obtained. 

## 7. Conclusions 

The study found that women in general have favorable attitudes and intentions toward screening for osteoporosis in a pharmacy, but favorable attitudes do not necessarily translate into decisions to use pharmacy testing in the future. Even though the HBM model constructs have been shown to be useful in the past for explaining patient's decision to screen for diseases like cancer, flu, TB, diabetes, and high blood pressure, and so forth, in our study, not all the model constructs are effectively related to the screening behavior. Women have relatively low perceptions of susceptibility and severity to osteoporosis despite higher levels of awareness regarding the disease. Perceptions of threat to the disease seem to have little impact on women's decision to screen for the disease. This is an area of concern, since the perceptions of susceptibility and severity to the disease are arguably the two key factors that shape and impact future preventive behavior, thereby having implications for the lower incidence of the disease itself. Overall, despite the lack of evidence showing stronger associations between HBM dimensions and the intended behavior, the HBM seems to work well in explaining the interrelationships among the model components and beliefs about pharmacy testing. 

## Figures and Tables

**Table 1:** Responses obtained from different research locations.

Locations	Questionnaires distributed	Questionnaires received	Usable questionnaires	Percentage
Senior center A	13	7	6	53.84%
Senior center B	19	7	6	36.84%
Senior center C	12	10	8	83.33%
Church	11	10	9	90.90%
St. John's Campus	117	83	80	71.79%

Overall	172	117	109	68.02%

**Table 2:** Study sample characteristics.

Sample characteristics^a ^	Frequency (*n* = 109) (%)
Ethnicity, no. (%)^b^	
Caucasians	62 (56.9%)
Asians	22 (20.2%)
African-American	11 (10.1%)
Age, no. (%)^b^	
18–30 years	41 (37.6%)
31–50 years	31 (28.4%)
>60 years	27 (24.8%)
Education, no. (%)^b ^	
Some years of college	31 (28.4%)
Four years of college	27 (24.8%)
Graduate degree	36 (33.0%)
Marital status, no. (%)^b^	
Single	43 (39.4%)
Married	51 (46.8%)
Widowed	11 (10.1%)
Health Insurance, no. (%)^b^	
Medicaid	6 (5.5%)
Medicare	14 (12.8%)
Private insurance	73 (67%)
Heard about BMD testing prior to this survey, no. (%)^b^	
Yes	77 (70.6%)
No	32 (29.4%)
Received BMD testing in a pharmacy, no. (%)^b^	
Yes	10 (9.2%)
No	99 (90.8%)

^a^Only top three major findings per demographic variable are reported in the table.

^b^Percentages do not sum to 100 due to missing data.

**Table 3:** Association between measures of outcomes and intention to screen.

	Likelihood of screening
Attitude toward screening in a pharmacy	0.553**
	0.000
Perceptions of severity	0.205*
	0.033
Perceptions of susceptibility	0.157
	0.102
Perceptions of benefits	0.311**
	0.001
Perceptions of barriers	−0.294**
	0.002
Perceptions of health motivation	0.033
	0.734

*Correlation is significant at the 0.05 level (2 tailed).

**Correlation is significant at the 0.01 level (2 tailed).

**Table 4:** Chi square analyses to test associations between respondents' sociodemographics and intention to screen.

Demographic factors	Likelihood of screening^a^		Excluded values	Chi square statistic	Significance
	Unlikely (*n*)	Likely (*n*)			
Age					
18–30 years	7	22	29	10.535	0.005
31–60 years	22	12			
>60 years	7	10			
Ethnicity					
Caucasian	25	11	29	3.089	0.079
Non-Caucasians	22	22			
Education					
Less than high school	6	4	29	1.512	0.680
Some years of college	10	11			
Four years of college	7	12			
Graduate degree	13	17			
Marital status					
Single/divorced/widowed	14	22	29	1.427	0.232
Married	23	21			
Insurance					
Private	25	30	42	0.082	0.775
Medicaid/Medicare	6	6			

^a^Total *N* = 109.

**Table 5:** ANOVA to examine differences in perceptions of health beliefs for women of different age groups.

Variables	*F*	Significance
Perceptions of susceptibility	0.981	0.378
Perceptions of seriousness	1.947	0.148
Perceptions of benefits	2.684	0.073
Perceptions of barriers	1.145	0.322
Attitude	10.169	0.000
Health motivation	0.558	0.574

In order to test difference of means between age groups, age was classified as 18–30 years (*n* = 41) 31–60 years (*n* = 41), and >60 years (*n* = 27).
